# AKT1 Transcriptomic Landscape in Breast Cancer Cells

**DOI:** 10.3390/cells11152290

**Published:** 2022-07-25

**Authors:** Bijesh George, Bin Gui, Rajeswari Raguraman, Aswathy Mary Paul, Harikrishna Nakshatri, Madhavan Radhakrishna Pillai, Rakesh Kumar

**Affiliations:** 1 Cancer Research Program, Rajiv Gandhi Centre for Biotechnology, Trivandrum 695014, India; bijeshgeorge@rgcb.res.in (B.G.); rajraghuraman@rgcb.res.in (R.R.); aswathym@rgcb.res.in (A.M.P.); 2 Graduate Degree Program, Manipal Academy of Higher Education, Manipal, Udupi 576104, India; 3 Biological Sciences, Ribon Therapeutics, Inc., Cambridge, MA 02140, USA; easter0731@gmail.com; 4 Department of Surgery, Indiana University of School of Medicine, Indianapolis, IN 46202, USA; hnakshat@iupui.edu; 5 Cancer Research Institute, Himalayan Institute of Medical Sciences, Swami Rama Himalayan University, Dehradun 248016, India; 6 Division of Hematology and Oncology, Department of Medicine, Rutgers New Jersey Medical School, Newark, NJ 07103, USA; 7 Department of Human and Molecular Genetics, School of Medicine, Virginia Commonwealth University, Richmond, VA 23298, USA

**Keywords:** breast cancer, AKT1, RNA-sequencing, transcriptome, emerging functions and targets, cancer therapeutics

## Abstract

Overexpression and hyperactivation of the serine/threonine protein kinase B (AKT) pathway is one of the most common cellular events in breast cancer progression. However, the nature of AKT1-specific genome-wide transcriptomic alterations in breast cancer cells and breast cancer remains unknown to this point. Here, we delineate the impact of selective AKT1 knock down using gene-specific siRNAs or inhibiting the AKT activity with a pan-AKT inhibitor VIII on the nature of transcriptomic changes in breast cancer cells using the genome-wide RNA-sequencing analysis. We found that changes in the cellular levels of AKT1 lead to changes in the levels of a set of differentially expressed genes and, in turn, imply resulting AKT1 cellular functions. In addition to an expected positive relationship between the status of AKT1 and co-expressed cellular genes, our study unexpectedly discovered an inherent role of AKT1 in inhibiting the expression of a subset of genes in both unstimulated and growth factor stimulated breast cancer cells. We found that depletion of AKT1 leads to upregulation of a subset of genes—many of which are also found to be downregulated in breast tumors with elevated high AKT1 as well as upregulated in breast tumors with no detectable AKT expression. Representative experimental validation studies in two breast cancer cell lines showed a reasonable concurrence between the expression data from the RNA-sequencing and qRT-PCR or data from ex vivo inhibition of AKT1 activity in cancer patient-derived cells. In brief, findings presented here provide a resource for further understanding of AKT1-dependent modulation of gene expression in breast cancer cells and broaden the scope and significance of AKT1 targets and their functions.

## 1. Introduction

Breast cancer (BC) is a polygenic and heterogeneous disease, which accounts for more than 24% of all cancers in women and about 684,996 deaths in 2020 [1]. The primary BC subtypes are stratified on the basis of the levels of estrogen receptor-alpha, progesterone receptor, and human epidermal growth factor 2 (HER2) receptor as well as on the basis of genomic, transcriptomic, epigenetic, morphological, and metabolic alterations. These alterations largely contribute to the noticed heterogeneity among more than 20 subtypes of breast cancers [2].

Cancer progression to more invasive phenotypes involves coordinated action of growth factors and oncogenes to counteract the activities of growth inhibitory pathways and tumor suppressors, in addition to other regulatory pathways. The process of oncogenesis is generally associated with dysregulated regulatory signaling, including mitogenic growth factors [3,4,5]. Mitogenic growth factors in conjunction with chromatin remodeling machinery (and other pathways) stimulate the proliferation, survival, motility, and invasive signaling pathways and resulting phenotypes [3,4,5,6,7]. One of such dysregulated signaling pathways in human cancer is the serine/threonine protein kinase B (AKT) pathway, which could be stimulated by multiple upstream molecules, i.e., insulin, platelet-derived growth factor (PDGF), insulin-like growth factor 1 (IGF1), epidermal growth factor (EGF), cytokines, nutrients, etc. [8]. The PI-3 kinase-AKT signaling pathway regulates cell cycle progression, survival, DNA repair, RNA export, differentiation, and tumorigenesis in several cancer cell types [4,8]. Accordingly, constitutive activation of this pathway has been also explored as a promising anticancer therapeutic strategy [9].

Activation of AKT signaling by growth factors, such as EGF, engages numerous downstream signaling cascades, leading to improved cell survival and proliferation in diverse cell types, including mammary epithelial cells [10,11]. The human AKT family of kinases consists of three distinct genes encoded on different loci, i.e., AKT1, AKT2, and AKT3, on chromosomes 14, 19, and 1, respectively. A large volume of initial studies in the field were conducted using AKT1 as a prototype of the AKT family, and conclusions drawn were initially presumed to also be implied for other AKT isoforms. However, a large body of work over the years involving either the gain- or loss-functions of AKT and AKT isoforms in the mouse and human model systems, respectively, has revealed differentiating biology of the AKT isoforms and their roles in the development and involution of the mammary gland as well as in the development and progression of breast cancer [12]. Previous studies have also shown that AKT1 mutations are found in ~1% of all cancers, and the most prevalent mutant AKT1(E17K) leads to its localization to the plasma membrane, invoking a consistent activation of AKT signaling in cancer cells [13,14].

All three AKT isoforms have been reported to be upregulated in human cancer and act as oncogenes and promote tumor proliferation at different levels [12,15,16]. In general, AKT1 knockdown leads to inhibition of tumor growth via blocking the cell-cycle progression and/or promoting apoptosis in breast cancer model systems [12,17,18]. Similarly, overexpression and/or constitutive activation of Akt1 in the mammary epithelial cells inhibits the pro-apoptotic signals as well as activates the survival signals to support the process of tumorigenesis [19,20]. Studies from transgenic mice suggested that Akt1 plays an important role in the initiation, development, and progression of breast tumors [20,21], whereas Akt2 has no major involvement in the process of tumor initiation but contributes to the process of tumor growth [20]. Consistent with these findings, hyperactivated AKT1 pathways are highly correlated with the initiation and development of breast cancer [21,22].

Results from knockout murine studies revealed that individual knockout of any one of the three isoforms was not lethal but contributed to growth retardation [23,24]. However, the double knockout murine studies involving Akt1 and Akt2 or Akt2 and Akt3 but not Akt1 and Akt3 were shown to be lethal in nature [25,26,27]. In the context of breast cancer, studies involving Akt1 knockout mice revealed that the loss of Akt1 suppresses ErbB2-induced mammary carcinogenesis [28] and mammary adenocarcinomas in mouse mammary tumor virus (MMTV)-ErbB2/neu, MMTV-polyoma middle T transgenic mice [29] and the growth of A2780 ovarian tumors in xenograft models [30]. AKT2 knockdown inhibited the chemotaxis of breast cancer cells [31], whereas knockdown of AKT3 resulted in reduced expression of HER2 and HER3 and upregulation of ER-alpha, resulting in an increased responsiveness of murine model cells to antiestrogen [32]. Ablation of AKT1 or AKT2 in murine breast cancer models and of AKT1 or AKT2 in human breast cell lines was associated with suppression of tumor progression and cell-cycle progression, increased apoptosis, and an overall reduced metastatic potential of target cells. In contrast, ablation of AKT3 has been shown to be associated with no major effect on the tumor growth but significantly decreases the tumorigenic potential of triple-negative breast cancer cells [33]. AKT2 has been shown to be involved in the maintenance of the tumorigenic characteristics of cells, as its knockdown was associated with tumor inhibition [33]. More recently, a circular AKT3 transcript has been shown to exert tumor suppressive function in glioblastoma cells, presumably by inhibiting the PI-3 kinase signaling [34]. In addition, AKT chemical inhibitors have been shown to inhibit the growth of model tumors through phosphorylation of downstream substrates in breast and other cancer cell types [35]. In addition, allosteric and competitive AKT inhibitors have been shown to prevent cancerous growth in a limited clinical study [15]. Because of structural similarities between the isoforms, many of the past experimental studies have utilized pan-AKT inhibitors such as MK2206, AZD5363, Ipatasertib, and perifosine [36,37].

The above studies suggest that in spite of a large body of work, the effect of the prototypic family member, AKT1, on the whole genome transcriptome in breast cancer cells remains unknown until this point, and hence, is examined in the present study.

## 2. Materials and Methods

Cell culture: Breast cancer cells MCF-7 and SKBR-3 were grown in Dulbecco’s modified eagle medium (DMEM) (Invitrogen, Waltham, MA, USA) containing 10% fetal bovine serum (FBS) (Invitrogen) and 1% penicillin-streptomycin antibiotic (Hi-media). The cells were maintained in a humidified atmosphere with 5% CO_2_ at 37 °C and used for experiments after they attained 70% confluence.

Transfection: Cells (4 × 10^5^ cells/mL) were seeded onto six-well plates and incubated for 24 h at 37 °C and later transfected with 50 nm AKT1 specific sure siRNA (Cat no. sc-29195) and non-specific siRNA (Cat no. sc-37007) from Santa Cruz Biotechnology, Inc., Dallas, TX, USA, in serum-free medium for 36 h. For EGF+ condition, cells were treated with 120 ng/mL of hEGF (Cat no. E9644, Sigma-Aldrich^®^ Solutions, St. Louis, MI, USA) for 8 h. Cells were harvested, and total RNA was isolated with the RNeasy extraction kit (Qiagen India Pvt Ltd., New Delhi, India).

RNA isolation: The total RNA from direct and indirect coculture assays was isolated with RNeasy kit (Qiagen India Pvt Ltd., New Delhi, India) following manufacturer’s instructions. The quality of RNA was checked with a Biospec nano spectrophotometer (Eppendorf, Hamburg, Germany), and 1 µg of total RNA was used for the cDNA conversion.

Single cell sequencing-based data analysis: AKT1, AKT2, and AKT3 expression data were extracted from single cell RNA-seq data of the healthy breast tissues using Loupe Browser (10X Genomics) as reported recently [38]. Markers used for cell type annotation are also described in the publication. The details of the samples and methods used to generate the referred single-cell sequencing atlas are described in Bhat-Nakshatri et al. [38].

RNA sequencing and resulting data processing: Total RNA samples were subjected to the whole transcriptome RNA-sequencing by Beckman Coulter Genomics, Newton, MA, USA. The vendor used the standard time-tested methodology for removing large and small ribosomal RNA, quality control, cDNA synthesis, DNA library preparation, paired-end sequencing with 2 × 100 bp using Illumina HiSeq 2000, read alignment to the reference hg19 (Ensembl GRCh37.75 build) genome using Tophat [39] version 2.0.9 in conjunction with Bowtie [40] version 1.0.0, quality control, and transcript assembly. Prior to mapping, reads are inspected and trimmed for adapter sequence with Flexbar [41] version 2.4. Thus, only reads not mapping to the transcriptome are attempted directly on the genome, allowing for prediction of novel exons, isoforms, and genes. Reads mapped to the transcriptome are documented with their genome-equivalent coordinates. ‘Proper’ read pairs either fall entirely within exons or hit adjacent exons. Singleton reads do not have their mate-read mapped on the genome due to sequence quality of the mate or to the incompleteness of the genome reference.

Read counting and differential expression analysis: Reads were counted using HTSeq-count [42] version 0.6.0, and multiple alignments were excluded. Gene counting was performed for genes and transcripts. Differential expression analysis was performed using DESeq package [43]. The samples were compared at gene level for all six experimental conditions to find the differentially expressed genes that are regulated by AKT isoforms. Together we have studied the transcription changes regulated by AKT gene by using pan-AKT inhibitor (Inhibitor VIII, a widely used AKT pan-inhibitor VIII) as well as its isoforms by knocking down AKT1 specific siRNA.

Complementary DNA (cDNA) conversion: The total RNA isolated from MCF 7 cells was converted to cDNA using the script cDNA synthesis kit (Bio-Rad). Briefly, 1 µg of total RNA was converted to DNA following manufacturer’s instructions. The mixture was incubated under the cycling conditions (25 °C for 5 min, 42 °C for 30 min, and 85 °C 95 for 5 min and 4 °C hold) in a PCR machine (Eppendorf) and were stored at −20 °C until use.

Quantitative reverse transcriptase polymerase chain reaction (qRT-PCR): The effect of knocking down AKT1 using siRNA or inhibiting AKT kinases by AKT inhibitor VIII on the levels of test genes in breast cancer cells, stimulated or unstimulated by EGF, was determined using qRT-PCR. The qRT-PCR reaction mixture containing 30 ng of cDNA was prepared with 5 µL SYBR-Green 2xmaster mix (TAKARA BIO INC., Kusatsu, Japan) and 0.4 µM each of forward and reverse primers (Sigma-Aldrich^®^ Solutions). The PCR reaction was carried out in an Applied Biosystem Quant Studio 7 plus real time PCR machine. Relative quantification of the gene expression (siNON v/s siAKT1) was determined using the 2-ΔΔCt method [44], and relative expression values (log 2-fold change (FC)) were normalized to GAPDH endogenous control values. The primer sequences for genes were commercially procured from Sigma Aldrich. The experiments were performed in triplicate for each sample.

Splice variation analysis: BAM files resulting from the processed RNA-Seq data alignment were analyzed for splice variations. BAM files were subjected to percent spliced-in (PSI) calculation using psi.sh as per the protocol described, and the exon inclusion counts were obtained [45,46]. Count files were fed to the DEXSeq [47] package in R with metadata to identify potential exon usage by each condition. DEXSeq provides differential expression analysis for a set of experimental conditions with a common denominator. Splicing events with *p*-value < 0.05 and *p*-adj < 0.1 were selected based on the higher exon usage coefficient to identify the highly abundant transcripts, and exons showing higher dispersion between AKT1 silenced samples and fold change are reported.

Comparative analysis with gene expression omnibus and TCGA datasets: Comparative analysis was performed for the differential expressed genes using AKT silenced datasets from GEO for the accession numbers GSE71900 [8] and GSE98078 [48]. Statistically significant (at least a fold change of 1.5 with a *p*-value < 0.05) genes were compared with the list of genes identified in our experiment to check the overlap, and the results are included. Gene expression analysis of breast cancer data are available from The Cancer Genome Atlas (TCGA) and Molecular Taxonomy of Breast Cancer International Consortium [49,50,51] samples. Samples are categorized into AKT1 high and low expressing samples using Onco-Query Language provided as per cBioPortal datasets [52,53].

Gene ontology and gene enrichment analysis: Gene ontology analysis was performed using the Funrich tool [54].

## 3. Results

### 3.1. Expression AKTs in Breast Cancer Cells

RNA interference (RNAi) technology coupled with gene expression analysis is widely used to map the regulatory network by inhibiting specific targeted mRNA. This approach is a powerful strategy to identify the transcriptomic variations, and, in turn, gain clues about the nature of the dysregulated pathways [55]. High throughput RNA sequencing allows us to capture the transcriptomic profile and compare test profiles to identify the alterations between distinct experimental settings.

**Figure 1:**
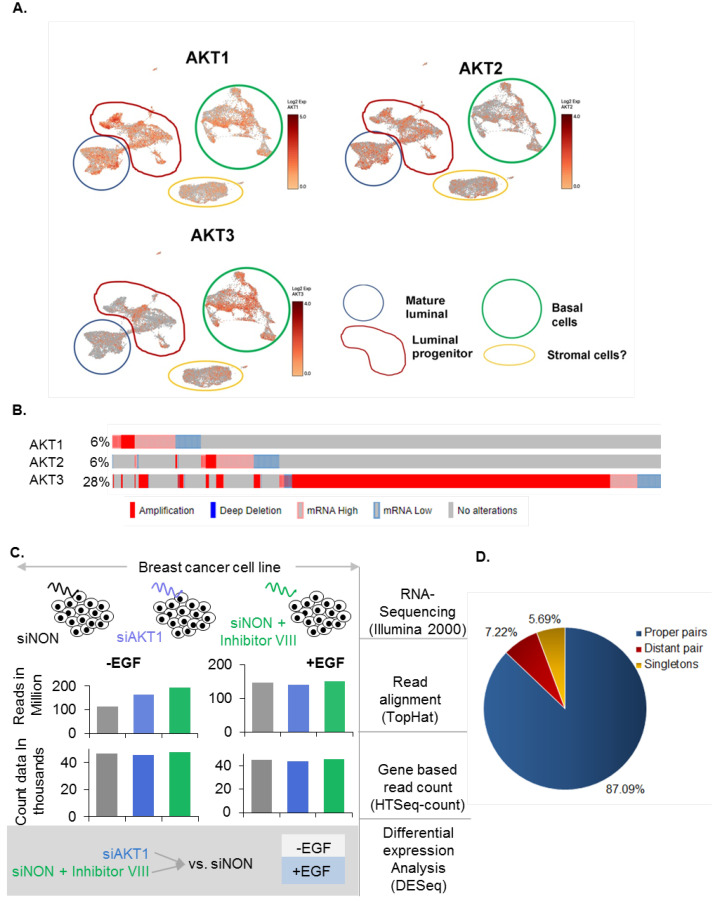
Expression of AKT isoforms and experimental strategy. (**A**) AKT1, AKT2, and AKT3 mRNA at the single-cell level of breast tissue. The image was generated using a recently published healthy breast atlas [38]. Expression of AKT1 and AKT2 was found to be widespread compared to AKT3. For example, AKT3 was expressed at a higher level in many subclusters of basal cells and in subcluster 4 of the luminal progenitor, but least in the mature luminal cells. The identity of the cells in yellow was suspected to be stromal in nature. (**B**) Expression of AKT isoforms in METABRIC datasets. (**C**) An overview of the analysis workflow, including the steps and the method followed to analyze the data and the numeric figures related to each step in the workflow. (**D**) Pie chart shows the average percentage of reads mapped to the human genome (hg19, Ensemble Grch37).

To understand the significance of AKT1 in the mammary epithelial cells, first we examined the status of AKT isoforms by single cell sequencing in major mammary epithelium cell types, i.e., basal cells, luminal progenitor, and mature luminal cells, isolated from healthy women [38]. We found that AKT1 is highly expressed in the basal cells, luminal progenitor, and mature luminal cells. In contrast, the expression of AKT2 somewhat overlapped with that of AKT1 (Figure 1A), suggesting that resulting phenotypes in mammary epithelial cells and, perhaps, in breast cancer, could be differentially affected by AKTs. We next examined the expression of AKT1, 2, and 3 in Breast Cancer METABRIC TCGA datasets [49,50,51,56] (Figure 1B). We found that each of AKT isoforms had a distinct overexpression pattern and that AKT1 and AKT2 are largely overexpressed as well as amplified, whereas AKT3 is predominantly amplified (and not overexpressed). Based on these observations and the results from the previous gain and loss of functional studies in the field, showing the role of AKT1 in the cell survival and growth regulation [12,15]—the focus of the present study—we decided to examine the impact of selective depletion of AKT1 and the inhibiting of the AKT’s activity by inhibitor VIII on the genome-wide transcriptome of breast cancer cells using breast cancer MCF-7 cells as a model system, as these cells express abundant levels of AKT1 and are widely used for a large number of genome-wide discovery studies. Results obtained were validated in MCF-7 and SKBR-3 breast cancer cell lines as well as in publicly available databases wherein human specimens were treated with the AKT inhibitor VIII.

### 3.2. Analysis of AKT1 Transcriptome in Breast Cancer Cells

The expression of endogenous AKT1 in MCF-7 cells was silenced using selective siRNAs directed against AKT1. In addition, in certain experiments, we used a pan-AKT inhibitor VIII, 5 nm for 30 min, which has been widely used to inhibit the activities of AKTs in multiple previous studies [57,58] (Supplementary Figure S1A). As the AKT pathway has been shown to be stimulated in cancer cells by growth factors, we chose to use EGF as a mitogen to stimulate the AKT1 pathway in MCF-7 cells. Cells were stimulated with or without 120 ng/mL EGF for 8 h after treating the cells with selective or control 50 nm siRNAs for 36 h (Figure 1C). Samples were prepared and subjected to paired-end RNA-sequencing [44], and we observed an average Pearson correlation of 0.93 between the replicates (Supplementary Table S1). Data analysis was performed using several commonly used, open-source algorithms and tools, schematically depicted in Figure 1C. An average of 78 million reads were generated for each sample, out of which an average of 87% of reads were aligned to human reference genome (Ensemble GRCh37.75 build, hg19) for each condition; 87.09% of reads were aligned as proper pairs, 7.22% were aligned as long pairs, and 5.69% were aligned as singletons (Figure 1D and Supplementary Table S2).

An average of 15,500 genes were identified per sample using Ensemble annotations. Genes with at least 10 aligned reads were considered for subsequent analyses (Supplementary Table S3), and a highly abundant 10 transcripts were considered as stable abundance and analyzed across the samples (Supplementary Table S4). Initial analysis on read mapping confirmed that the read mapping was proper and thus excluded the possibility in the variation in read distribution among the treatment conditions. In brief, these studies accurately mapped the high quality paired end reads to the human genome and, thus, appropriateness of the read depth and coverage for further analysis.

### 3.3. Influence of AKT1 on the Status of Growth Factor Induced Genes

**Figure 2:**
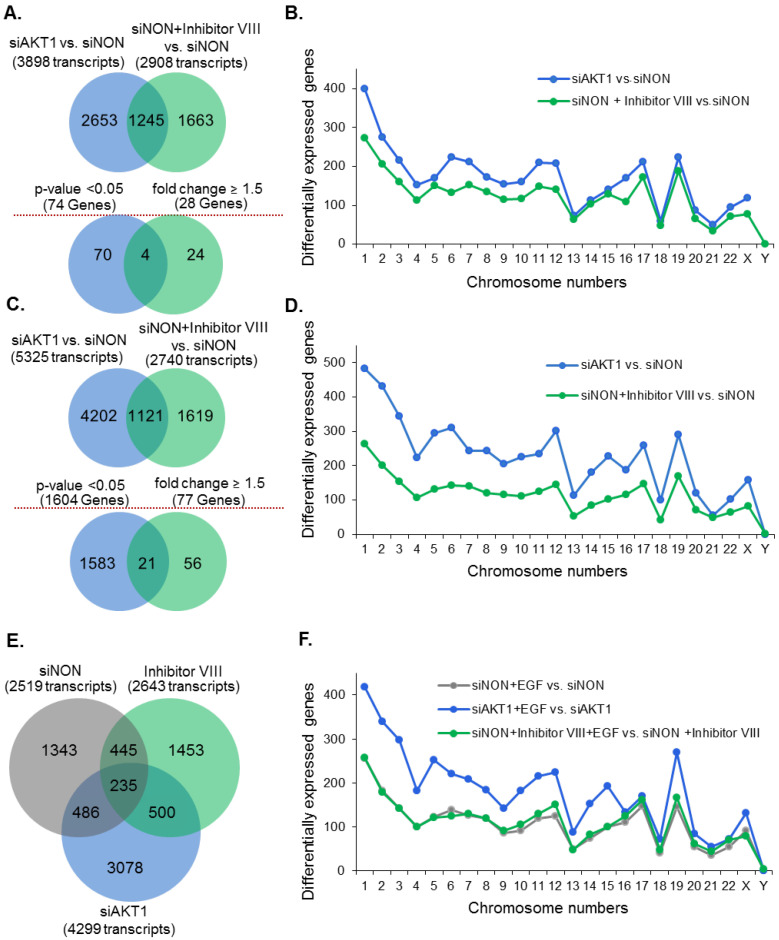
Effect of AKT1 on the status of differentially expressed genes in breast cancer cells. (**A**) Venn diagram showing the shared and uniquely expressed differentially expressed genes upon depletion of AKT1 or treatment with AKT inhibitor VIII as compared to cells treated with siNON. The total number of differentially expressed transcripts with 2-fold change are represented in parentheses. Results of comparative analysis of statistically significant (*p*-value < 0.05 and fold change > 1.5) differentially expressed genes are shown in the lower panels; (**B**) ≥chromosome-wise distribution of differentially expressed transcripts corresponding to the preceding upper panel (**A**); (**C**) Venn diagram showing the comparison of the number of differentially expressed transcripts with 2-fold change in EGF-stimulated breast cancer cells. Results of comparative analysis of statistically significant (*p*-value < 0.05 and fold change > 1.5) differentially expressed genes are shown in the lower panels; (**D**) chromosome-wise distribution of transcripts corresponding to genes in upper panel (**C**); (**E**) genes altered (>2-fold change) in the indicated experimental conditions in EGF-stimulated breast cancer cells; and (**F**) chromosome-wise distribution of differentially expressed genes in EGF-stimulated breast cancer cells in Panel (**E**).

Differentially expressed genes were obtained for each experimental condition with respect to control and with and without EGF stimulation. To assess the effectiveness of the selective AKT1-siRNA used, we first determined the levels of AKT1 mRNA (and AKT2 and AKT3 mRNA as controls) in the processed datasets, in addition to initial examination of the AKT1 protein (Supplementary Figure S1A). As expected, use of AKT1-siRNA was accompanied by a reduced expression of AKT1 (not AKT3 as a negative control) (Supplementary Figure S1B). For obtaining an overall larger view of gene distribution among experimental conditions and for performing an initial assessment of AKT1 isoform specific changes in the transcriptome, the comparison of the differentially expressed genes was performed before applying the statistical threshold in Figure 2. This was followed by implementation of the quality control measures to select differentially expressed genes with at least a 1.5-fold change and a *p*-value less than 0.05 for further analysis. Statistically significant genes with *p*-value < 0.05 and >1.5-fold change were identified using a negative binomial test. A total of 3898 and 2908 genes were found to be differentially expressed, respectively, for AKT1 knockdown and cells treated with inhibitor VIII with at least a 2-fold change over the cells treated with the control siRNA (siNON). Comparative analysis of differentially expressed genes found sets of 2653 and 1663 genes to be uniquely regulated by siAKT1 and Inhibitor VIII (AKT-VIII), respectively, in unstimulated breast cancer cells (Figure 2A). Upon EGF stimulation, these affected gene numbers were changed to 5325 (siAKT1) and 2740 (AKT-VIII) differentially expressed genes. As the goal of the study was to determine the nature of AKT1-dependent modulation of transcriptome, we found a total of 4202 and 1619 genes were uniquely regulated by siAKT1 and inhibitor VIII, respectively (Figure 2C). The number of differentially expressed genes in each chromosome was analyzed to observe the choice of the preferred target gene genomic loci. We noticed that chromosomes 1 and 19 represent relatively higher fractions of altered genes in both unstimulated and EGF-stimulated breast cancer cells, whereas differentially expressed genes on chromosome 2 were observed only upon EGF stimulation (Figure 2C,D). In brief, we observed that AKT1 could regulate specific set of genes and thus could influence the nature of breast cancer transcriptome.

### 3.4. EGF Modulation of AKT-Dependent Transcriptome

As we were interested in understanding the effect of EGF stimulation of AKT1-dependent transcriptome, we found that a total of 2519, 4299, and 2643 genes were altered, with at least 2-fold change in expression, in cells treated with siNON, siAKT1, and VIII, respectively. Among these differentially expressed genes, 1343, 3078, and 1453 genes were found to be unique to the referred experimental conditions (Figure 2E). Chromosome-wise distribution showed that EGF stimulation in the absence of AKT1 alters a higher number of genes across the genome (Figure 2F).

**Figure 3:**
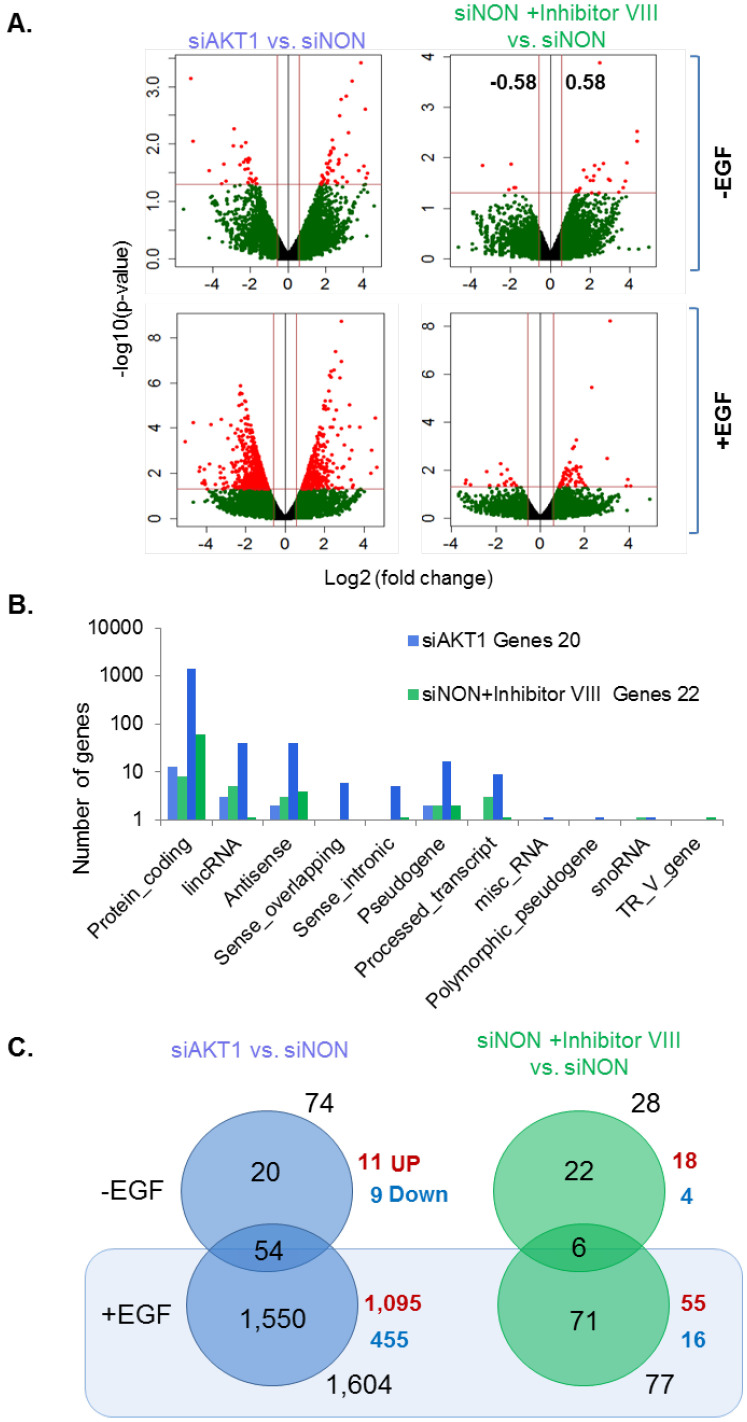
Status of statistically significant differentially expressed genes. (**A**) Statistical distribution of differentially expressed genes; *p*-value 0.05 threshold is marked on the Y-axis (red) and 2-fold change (green) are marked on the X-axis; (**B**) overall distribution of the coding potential of the statistically significant genes across experimental conditions; and (**C**) pictorial representation of differentially expressed genes across various experimental conditions.

We next analyzed the fold-change distributions against statistical significance, i.e., differentially expressed genes with at least 1.5-fold change with *p*-value < 0.05 for further analysis (Figure 3A). Differentially expressed genes were categorized based on the coding potential; the protein coding genes were found highly altered followed by lncRNAs and antisense RNAs (Figure 3B, Supplementary Table S5). A set of 1624 genes were found to be significantly affected by the knockdown of AKT1 with respect to siNON in the absence or presence of EGF stimulation (Figure 3C). In brief, we describe a set of differentially expressed genes which are preferentially modulated by the levels of AKT1.

### 3.5. Identification of AKT1 Specific Regulatory Pathways

**Figure 4:**
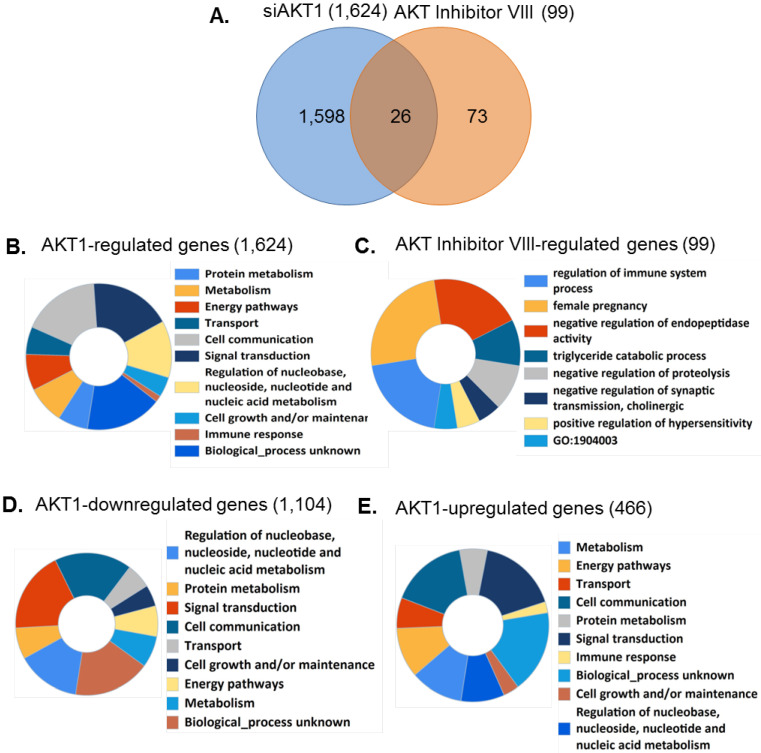
Pathway and functional analysis of AKT1-dependent differentially altered genes. (**A**) Venn diagram showing the shared and unique genes regulated by AKT1 and/or AKT inhibitor VIII; (**B**) doughnut chart showing the functional annotation of differentially expressed genes upon AKT1 silencing using siAKT1; (**C**) doughnut chart showing the functional annotation of differentially expressed genes upon using the Pan-AKT Inhibitor VIII; (**D**) doughnut chart showing the functional annotation of downregulated genes upon AKT1 silencing; and (**E**) doughnut chart showing the functional annotation of upregulated genes upon AKT1 silencing.

We next compared the genes affected by the status of AKT1 and identified a total of 1624 genes to be regulated by AKT1 (Figure 4A). Gene ontology analysis of 1624 genes showed an enrichment of these genes in protein metabolism, metabolism, energy pathways, cell growth and/or maintenance, transport etc. (Figure 4B, Supplementary File S2). A set of 99 AKT regulated genes were functionally involved in regulation of the immune system process, female pregnancy, negative regulation of endopeptidase activity, triglyceride catabolic process, etc. (Figure 4D, Supplementary File S2). Comparative analysis of functions of AKT1 regulated genes versus genes regulated when all the AKTs were inhibited by the Pan-AKT Inhibitor VIII revealed sharing of only six predicted functions, namely, signal transduction, immune response, cell cycle, cell adhesion, cell proliferation, and cell differentiation (Supplementary Figure S2, Supplementary File S2). A set of 1104 genes found downregulated were functionally involved in protein metabolism, metabolism, energy pathways, cell growth and/or maintenance, transport, cell communication, signal transduction, etc. (Figure 4D, Supplementary File S2). Interestingly, 466 AKT-dependent upregulated genes were also functionally annotated and found to be largely involved in the biological processes like protein metabolism, metabolism, energy pathways, cell growth and/or maintenance, transport, cell communication, etc. (Figure 4E, Supplementary File S2). A total of 466 AKT-knockdown associated upregulated genes might be important, as these genes were presumably inhibited by the presence of an active AKT pathway. An overall gene ontology analysis [59,60,61] of these 466 genes showed that a large number of these genes are involved in metabolic related functions (Figure 4D). Among the 466 genes upregulated in the absence of AKT1 (and hence, these genes are expected to be downregulated by AKT1), 25 genes are downregulated in breast cancer with high AKT1 expression [62]. In brief, our study strengthens the notion of AKT1-specific functions and discovered an unexpected role of AKT1 signaling in inhibitory transcriptomic changes.

### 3.6. Validation of AKT1 Regulated Significant Genes

**Figure 5:**
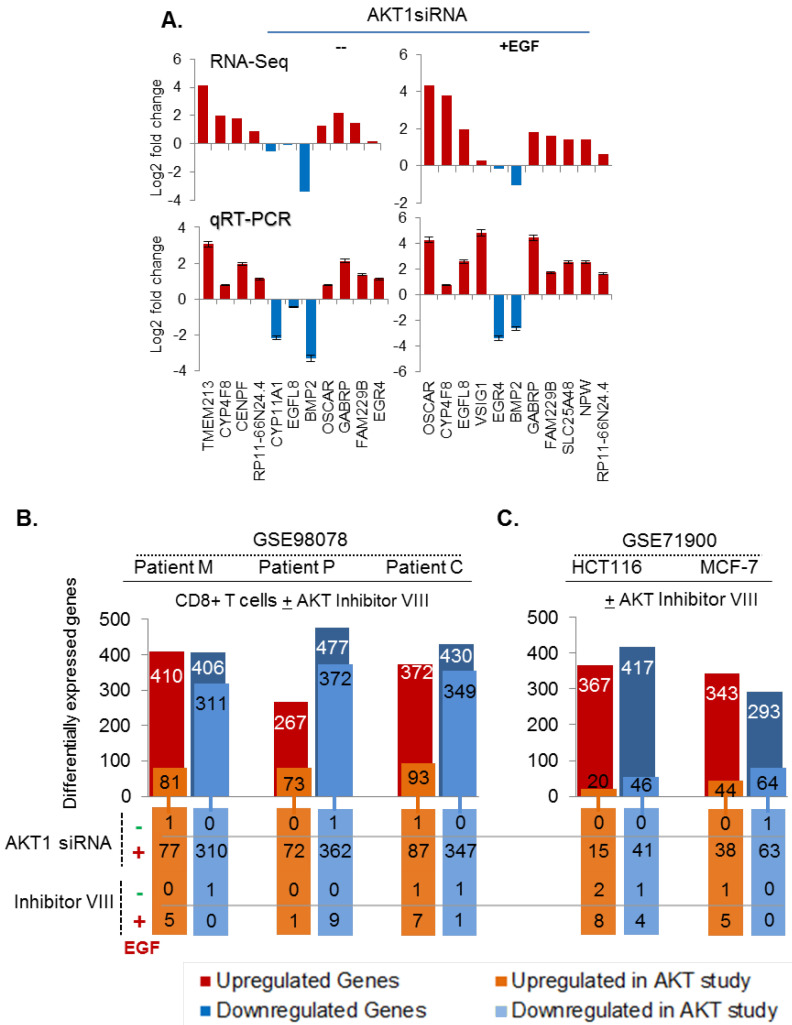
Validation of AKT-dependent modulation of transcriptomes. (**A**) Examples of genes with a similar expression pattern in the RNA-seq data and RT-PCR validation studies; (**B**) comparative analysis of RNA sequencing data presented here with AKT inhibitor VIII-modulated transcriptome from the accession number GSE98078. The numbers on the top of the bar show the overlap with upregulated and downregulated genes as compared to the individual sample; the lower part of the diagram shows the number of gene with unique overlap with AKT inhibitor VIII treated samples with and without EGF stimulation; and (**C**) the comparative analysis of the RNA-seq data presented here with the effect of AKT inhibitor VIII on the gene expression in colorectal cancer HCT116 and breast cancer MCF-7 cells under the accession number GSE71900. Only the genes showing at least 1.5 fold change with a *p*-value < 0.05 are considered from all the studies.

Next, we validated a set of selected genes of interest from the RNA-seq analysis using qRT-PCR in two different breast cancer MCF-7 and SKBR-3 cell lines. First, MCF-7 cells were treated with siAKT1 or control siNON, followed by stimulation with or without EGF for 8 h. In general, we noticed a similar trend of increased or reduced expression of 11 tested transcripts, out of 21 selected genes, belonging to the pathways of interest to the laboratory, between the RNA-sequencing and qRT-PCR results in cells with siAKT1 (Figure 5A, Supplementary Figure S3) and among them 7 (without EGF) and 6 (EGF stimulated samples) genes were also found to exhibit the same pattern of expression in another breast cancer SKBR3 cell line (Supplementary Figure S3).

To further validate the noticed modulation of AKT1 status-dependent transcriptome in patient-derived biomaterial, we attempted to search for datasets from human cancer or other studies. We found two AKT-silencing based transcriptome datasets from the Gene Expression Omnibus (GEO). The first dataset reported mRNA expression profiles of CD8+ T cells, derived from an unrelated study involving three acute lymphocytic leukemia (ALL) patients—designated as patient M, P, and C, and ex vivo treated with a pan-AKT inhibitor VIII [63]. We observed an overlap of 816 (410 upregulated, 406 downregulated), 744 (267 upregulated, 477 downregulated), 802 (372 upregulated, 430 downregulated) genes between the results from MCF-7 cells and patients M, P, and C, respectively (Figure 5B). When we analyzed the status of upregulated and downregulated genes individually upon silencing AKT1, we observed a higher number of overlaps among the downregulated genes (Figure 5C, blue bars). Upon comparing the levels of overlapped genes between AKT1-silenced MCF-7 cells with the AKT inhibitor VIII-treated CD8+ T cells (Figure 5B, lower panel), we observed a substantial overlap between two differentially regulated gene sets, especially under conditions of EGF stimulation. For example, we noticed an overlap of 77 upregulated genes between EGF-stimulated MCF-7 cells with siAKT1 and 81 upregulated genes in cells from patient M. Similarly, there was an overlap of 310 downregulated genes between EGF-stimulated MCF-7 cells with siAKT1 and 311 downregulated genes in patient M-derived cells treated with pan-AKT inhibitor. Using the second dataset, we performed a comparative analysis of our results with previously reported differentially expressed genes in AKT inhibitor VIII-treated HCT116 colorectal cancer cells and MCF-7 breast cancer cells [8]. As illustrated in Figure 5C, cancer cell lines also exhibited a similar pattern in the levels of differentially expressed genes with that of patients (Figure 5C, blue bars).

As elucidated in Figure 5A, one of the interesting observations of the present study is it revealed an unexpected role of active AKT1 signaling in inhibiting the expression of certain genes through an undefined mechanism at the moment—as silencing of AKT1 resulted in elevated expression of such genes. Examples of such validated genes include transmembrane protein 213 (TMEM213), upregulated in cells treated with siAKT1; Cytochrome P450 Family 4 Subfamily F Member 8 (CYP4F8), Gamma-aminobutyric acid receptor subunit pi (GABRP) and osteoclast-associated immunoglobulin-like receptor (OSCAR)—upregulated in cells treated with siAKT1, etc. Consistent with these observations, we noticed that overexpression of AKT1 mRNA in breast tumors in the TCGA dataset is generally accompanied by a substantial reduction in the levels of TMEN213, VS1G1, CYP4F8, HAS3, and OSCAR (Supplementary Figure S4). In brief, these results validated the notion that the nature of AKT1-dependent transcriptomic shows changes in cellular and patient-derived ex vivo cellular models and revealed a set of gene expression that might be negatively affected by AKT1 signaling.

### 3.7. Influence of the Endogenous Status of AKT1 on Splice Variation

Transcript variants are the number of different mRNAs reported for a single gene from a single transcription start site (TSS) that might contribute to genomic heterogenicity [62,64] and are also reported to be important to splicing in breast cancer [65]. AKT signaling is known to be involved in alternative splicing through phosphorylation of its downstream substrates and, in turn, modulation of the post-transcriptional regulation of target genes [66,67]. Having observed an effect of the selective depletion of AKT1 on the genome-wide transcriptome of MCF-7 cells, we reasoned that AKT, being a kinase, might also influence the functionality of the splicing machinery through yet-to-be defined mechanisms and, hence, lead to splice variants. We next attempted to examine the status of splice variance among highly abundant transcripts across different experimental conditions in the presence or absence of EGF stimulation. Splice variation was estimated using the number of unique reads mapped to the spliced region when compared to read abundance for each exon for a single gene.

**Figure 6:**
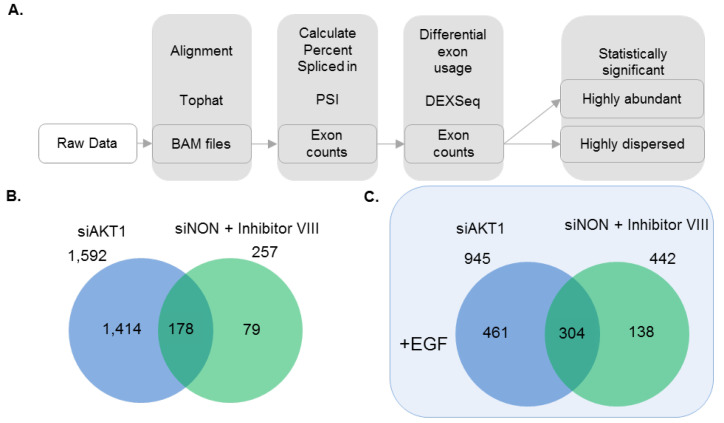
Identification of splice variants in AKT1-dependent transcriptome. (**A**) Tools and pipeline to identify the splice variants; and (**B**,**C**) comparative analysis of splice variants identified in each experimental conditions without (**B**) or with (**C**) EGF stimulation. Only the spliced genes showing at least 2-fold change with a *p*-value < 0.05 are considered from all the studies.

Splice variation analysis was performed on transcriptome of AKT1 silenced MCF-7 cells and compared to control siNON with and without EGF stimulation using publicly available tools and algorithms (Figure 6A). A total of 3236 statistically significant (*p*-value < 0.05 and fold change > 2) splicing events were identified from all the comparisons: the splicing was counted only if more than 10 reads mapped to the target region. We found that AKT1 knockdown exhibited an overall higher number of splicing events with a similar trend of expression than that of differential expression of the full-length transcripts. A total of 1592 splice variations were identified in MCF-7 cells with AKT1 knockdown and 257 splice variances in cells treated with Inhibitor VIII in cells without EGF stimulation. Interestingly, EGF stimulation of breast cancer cells leads to 945 splice variances in cells with AKT1 knockdown and 442 splice variances in Inhibitor VIII-treated cells. These findings suggested the status of AKT1 might have an effect on the magnitude of influence on the genome-wide splice variance (Figure 6B,C). We next analyzed the top 10 ranked exon splicing events that were either highly abundant or dispersed between the cells with AKT1 knockdown. One of such examples is the ribosomal protein family, of which six out of ten highly abundant splice variations are identified to be affected by the status of AKT1 (this study, Supplementary Tables S6 and S7).

### 3.8. Top 10 Highly Dispersed Splicing Events between the AKT1 Knockdown

**Figure 7:**
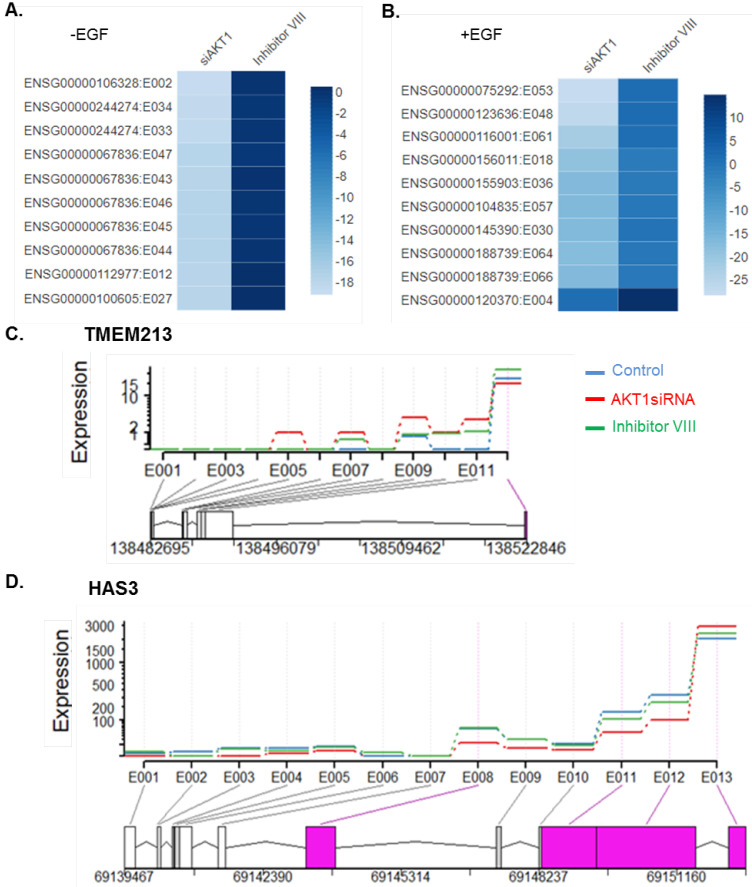
Highly dispersed splicing events observed between AKT1 silenced cells upon EGF stimulation. (**A**,**B**) Top 10 differential spliced variants in AKT1 silenced samples with or without EGF stimulation; (**C**) exon-based splicing of TMEM213 showing differential splicing of exon 12 under indicated experimental conditions; and (**D**) exon-based splicing pattern of HAS3 showing differential splicing of exon 8, 11, 12, and 13. Spliced exons are denoted in pink.

We found 13 examples of spliced variants downregulated upon AKT1 silencing. Splice variants related to four genes (DBNDD2, DAP, ITPK1, and ROGDI) previously reported upregulated in breast/other human cancers were found downregulated in AKT1 silenced samples compared to control (siNoN) (Supplementary Table S6). Data in Figure 7A show the expression levels of top 10 spliced variants. For example, the Fascin-3 variant FSCN3:2/exon 2 was reported differentially upregulated in metastasis [68]. Dysbindin domain-containing protein 2 variant DBNDD2:34 that was upregulated in BRCA1 mutated breast cancer [63] was found downregulated in the AKT1 silenced sample compared to control (siNoN). Inositol-tetrakisphosphate 1-kinase variant ITPK1:27 was found over expressed in breast cancer [69] but was also found downregulated in the AKT1 silenced sample compared to control (siNoN). Protein rogdi homolog variant ROGDI:43–47, upregulated and reported as a target molecule in cervical cancer [70], was found downregulated in the AKT1 silenced sample compared to control (siNoN). However, upon EGF stimulation, we observed 10 examples of spliced variants downregulated upon AKT1 silencing, among them many were reported with an alteration related to breast/human cancers (Figure 7B, Supplementary Table S7). For example, Nucleolysin TIA-1 isoform p40 variant TIA1:61 was found downregulated in the AKT1 silenced sample compared to control (siNoN), which was reported downregulated in human cancers [71]. PH and SEC7 domain-containing protein 3 variant PSD3:18 found downregulated in the AKT1 silenced sample compared to control was reported downregulated in breast cancer [72]. Interestingly, we also observed examples of AKT1-dependent splicing of two validated genes, TMEM213 and HAS3.

## 4. Discussion

Many extrinsic signals influence the pro-survival and invasive phenotypes of cancer cells by stimulating the AKT signaling pathway and its downstream cellular processes feeding into cancer phenotypes. In addition, the functionality of signal-dependent cellular events is profoundly determined by the functionality of transcriptomic heterogenicity, which, in part, is influenced by differencing splicing in addition to other regulatory steps of transcriptomic and post-transcriptional mechanisms of gene expression. In this context, here we uncovered the effect of the status of AKT1 as well as inhibition of AKT’s kinase activity on the genome-wide transcriptomic and differential splicing events in breast cancer cells. Our experimental strategy involved selectively knocking down the endogenous AKT1 as well as treating the model breast cancer cells with a pan-AKT activity Inhibitor VIII. This was followed by stimulation of cells with EGF before subjecting them to genome-wide RNA-sequencing.

We observed that silencing of the endogenous AKT1 and/or inhibiting AKTs could alter the expression of up- and downregulated differentially expressed transcripts in breast cancer cells. As AKT1 has been shown to be overexpressed and/or hyperactivated in breast tumors, our results imply that many downstream phenotypic effects of AKT are not merely mediated by AKT1-signaling dependent phosphorylation of its direct substrates but also by genomic effects of AKT1. It remains unclear how exactly AKT1 contributes to the noticed genomic changes. The highest population of AKT status-dependent gene alterations were found on chromosome 1 (the longest chromosome with the highest number of genes) and chromosome 19. As chromosome 19 has been shown to exhibit extremely high incidence of loss of heterozygosity linked with breast cancer metastasis [73], it is interesting to observe that AKT1 signaling also preferentially modulates the expression of genes on chromosome 19—the underlying basis of which remains unknown at this time. As cancer cells are exposed to a variety of mitogenic growth factors, one of our experimental strategies was to also reveal the nature of transcriptomic changes in cells stimulated with epidermal growth factor. We noticed that only a portion of AKT-responsive transcripts undergoes further alterations in its expression in growth-factor stimulated cells, presumably due to stimulated hyperactivated AKT1 signaling by EGF. These findings imply that the nature of AKT1-responsive pathways is not only affected by the levels of AKT1 transcripts but also by the presence of mitogenic signals feeding into AKT1 signaling.

As expected, we observed a positive relationship between the status of AKT1 expression and many differentially expressed genes related to cellular processes. This could be attributed to the ability of AKT1 kinase to phosphorylate its substrate and/or cascade effects on the transcriptome. The top ten AKT1-dependent highly abundant transcripts identified in the present study—TFF1 (ENST00000291527), EEF2 (ENST00000309311), SCD (ENST00000370355), SPTSSB (ENST00000359175), KRT81 (ENST00000327741), LAPTM4A (ENST00000175091), NUCKS1 (ENST00000367142), LAPTM4B (ENST00000445593), PERP (ENST00000421351), and KRT19 (ENST00000361566)—are also found to be upregulated in human cancers, and nine of them are highly significant cancer-associated genes in breast cancer (Supplementary Table S4). This suggests that biological effects of generally noticed AKT1-hyperactivation in human cancers, including breast cancer, might be impacted by co-overexpression of AKT1 status-dependent expression of a subset of genes. To examine the validity of this hypothesis, we performed a multivariate analysis of AKT1 overexpression in conjunction with 10 highly abundant AKT-dependent transcripts on the overall disease-free survival of patients with breast tumors. We found that, indeed, co-overexpression of test genes further shortens the duration of overall survival of patients as compared to AKT1 alone (Supplementary Figure S5).

As many components of the cellular splicing machinery are phosphoproteins and the process of differential splicing has been shown to be regulated by upstream signaling [73], the present study also sheds new light on the effect of the AKT1 status on the splicing events, which, in turn, also contributes to tumor heterogeneity. Our results revealed that the cellular status of AKT1 might determine the magnitude of splice variance (Figure 6). Interestingly, many of the examples of the top 10 highly dispersed splicing events affected by the status of AKT1 have been previously implicated in cancerous phenotypes [63,68,69,70,71,72,74,75,76,77,78,79,80,81]. Though not in the top 10, we also found examples of AKT1-dependent transcripts that also underwent differential splicing in a manner dependent on the presence or silencing of AKT1. Examples of such genes include TMEM213, and HAS3 (Figure 7C,D).

Our observation that the absence of AKT1 leads to upregulation of about 466 genes represents another notable unexpected finding, as it uncovered a potential role of AKT1 signaling on the expression of a subset of genes—the mechanism of which is yet to be defined. A broader significance of this finding might be that 25 of such loss-of-AKT1-associated upregulated genes, implying that these might be normally inhibited by AKT1, have been shown to be downregulated in breast cancer [62]. Examples of the noticed unexpected upregulation of candidate genes upon AKT1 silencing include: cytochrome P450 family 4 subfamily F member 8 (CYP4F8)—involved in drug metabolism and biosynthesis of lipids and cholesterol; osteoclast-associated receptor (OSCAR)—involved in adaptive and innate immunity; and two poorly studied genes—transmembrane protein 213 (TMEM213) and V-Set and Immunoglobulin Domain Containing 1 (VSIG1) (see below).

Transmembrane protein 213 (TMEM213) is a poorly studied protein-coding gene with a predicted protein localization in the endoplasmic reticulum. TMEM213 has been shown to be downregulated in clear cell renal cell carcinoma with a predicted association with invasion and metastasis [82], whereas it is upregulated in lung adenocarcinoma and contributes to a longer survival of patients [83]. Bioinformatic studies have largely linked TMEM213 to pathways with roles in drug metabolism and transporters [83]. The levels of TMEM213 were found to be upregulated upon silencing AKT1 (this study), and an inverse correlation exists between the levels of AKT1 and TMEM213 in breast tumors (Supplementary Figure S4a). Additionally, overexpression of TMEM213 in breast tumors was found to be associated with better survival (Supplementary Figure S4b); we suggest that the generally observed increased expression and/or hyperactivation of PI-3 kinase/AKT signaling might impair the expression of TMEM213 and the resulting role in cancer progression.

V-Set and Immunoglobulin Domain Containing 1 (VSIG1) protein is a recently discovered member of the junctional adhesion family and has been widely dysregulated in human cancer. Earlier studies suggest that the levels of VSIG1 have been implicated in pro-metastatic and EMT: its reduced expression correlates with a poor prognosis [84] and differentiation [85] of certain cancer types [85,86]. As expression of VSIG1 might be repressed by AKT expression/signaling (this study) and the fact that VSIG1 is predicted to be localized in the plasma membrane (https://www.genecards.org/cgi-bin/carddisp.pl?gene=VSIG1, accessed on 12 November 2021); these findings raise the possibility that some of the recognized cancerous phenotypes of AKT1 might be mediated by its influence on the levels of VSIG1 through an undefined mechanism at this point.

In brief, results presented here shed new insights on the significance of AKT1 signaling on the genome-wide transcriptome and differential splicing in breast cancer cells. In addition to broadening the scope of AKT1-dependent positive regulation of gene expression, our study unexpectedly discovered that an active AKT1 signaling could also inhibit gene expression—many of which are widely known to be downregulated during cancer progression. These initial findings have raised several follow-up issues, including further experimental validation in multiple cellular models and delineating the fine mechanistic details through which AKT1 contributes to gene expression [49,50,51,52,53,63,68,69,70,71,72,77,86,87,88,89,90,91,92,93].

## Data Availability

The data presented in this study are available in Supplementary material and sequencing raw files deposited to the NCBI SRA and available under the accession PRJNA859811.

## Supplementary Materials

The following supporting information can be downloaded at: https://www.mdpi.com/article/10.3390/cells11152290/s1, Figure S1: Expression levels of AKTs; Figure S2: Comparative analysis of functions regulated by AKT1 regulated genes versus the functions inhibited by pan-AKT inhibitor VIII; Figure S3: Status of the fold-change values from the RNA-sequencing and RT-PCR assays for selected genes using MCF-7 and SKBR-3 cell lines; Figure S4: The expression levels of selected genes in breast cancer datasets along with the changes in patient survival; Figure S5: Gene expression and survival analysis of select highly abundant genes; Table S1: The Pearson correlation coefficient between the replicates; Table S2: Details of the reads mapped to the genes for each sample; Table S3: The number of genes identified from different experimental conditions; Table S4: The number of 10 highly abundant transcripts across experimental conditions; Table S5: The number of potential differentially expressed genes according to their coding potential; Table S6: The 10 selected and highly spliced genes compared to control when AKTs are silenced from samples without EGF stimulation; Table S7: The 10 selected and highly spliced genes compared to control when AKTs are silenced from EGF stimulated samples; Supplementary File S2: Complete results of the functional analysis of differentially expressed genes.
