# Supplementary material for: AKT1 Transcriptomic Landscape in Breast Cancer Cells
Source: Cells. 2022 Jul 25;11(15):2290. doi: 10.3390/cells11152290 (PMC9332453; doi:10.3390/cells11152290)
Supplement: Supplementary file 1 [file cells-11-02290-s001.zip › Supplementary File S1.pdf]

Supplementary File S1

AKT1 Transcriptomic Landscape in Breast Cancer Cells

Bijesh George, Bin Gui, Rajeswari Raghuraman, Aswathy Mary Paul, Harikrishna Nakshatri, Madhavan Radhakrishna Pillai, and Rakesh Kumar

Sl. No	Sample Name	Treatment	Correlation
1	Inhibitor VIII R1	siNON + Inhibitor VIII	0.95863
2	Inhibitor VIII R2	siNON + Inhibitor VIII	
3	AKT1 R1	siAKT1	0.87775
4	AKT1 R2	siAKT1	
7	Control R1	siNON	0.96161
8	Control R2	siNON	
9	AKT1 EGF R1	siAKT1 + EGF	0.98277
10	AKT1 EGF R2	siAKT1 + EGF	
13	Control EGF R1	siNON + EGF	0.98385
14	Control EGF R2	siNON + EGF	
15	InhibitorVIII EGF R1	siNON + Inhibitor VIII + EGF	0.98095
16	Inhibitor VIII EGF R2	siNON + Inhibitor VIII + EGF	

Supplementary Table S1: The Pearson correlation coefficient between the replicates.

Condition	QC passed reads	GC	raw reads mapped	pairs mapped	proper pairs	singletons
Control R1	780,13,338	50%	792,26,114	94.30%	88%	5.70%
Control EGF R1	764,95,166	49%	781,63,657	94.80%	91%	5.20%
Inhibitor VIII EGF R1	746,83,608	51%	756,40,477	93.70%	88.20%	6.30%
InhibitorVIII R1	764,89,640	51%	772,70,933	94.10%	90.90%	5.90%
siAKT1 R1	766,53,294	51%	772,99,453	93.60%	89.50%	6.40%
siAKT1 EGF R1	658,95,066	51%	673,02,992	94.70%	88.50%	5.30%
Control R2	646,80,720	48%	655,57,296	94.80%	86.10%	5.20%
Control EGF R2	681,97,242	49%	694,53,312	93.90%	83.40%	6.10%
Inhibitor VIII EGF R2	760,89,630	49%	762,57,421	93.50%	84.60%	6.50%
InhibitorVIII R2	1146,18,348	49%	1169,39,293	94.90%	84.50%	5.10%
AKT1 R2	849,63,954	48%	864,92,961	94.70%	86.40%	5.30%
AKT1 EGF R2	746,82,246	50%	753,64,429	93.70%	86.20%	6.30%

Supplementary Table S2: Details of the reads mapped to the genes for each sample.

**Sample**	**Gene counts**	
	Genes with at least 10 Reads mapped	Total gene count
Control R1	15739	22569
Control + EGF R1	15872	22422
Inhibitor VIII+ EGF R1	15616	22302
InhibitorVIII R1	15691	21851
siAKT1 R1	15343	21455
siAKT1+ EGF R1	15259	21970
Control R2	15869	24880
Control+ EGF R2	15868	23324
Inhibitor VIII+ EGF R2	16237	24110
InhibitorVIII R2	17516	26589
siAKT1 R2	16827	25001
siAKT1+ EGF R2	15874	22882

Supplementary Table S3: The number of genes identified from different experimental conditions.

Gene	Abundant Transcript	Chr	Start	End	Number of Transcripts
TFF1	ENST00000291527	chr21	43782391	43786703	1
EEF2	ENST00000309311	chr19	3976054	3985467	7
SCD	ENST00000370355	chr10	1.02E+08	102124591	1
SPTSSB	ENST00000359175	chr3	1.61E+08	161090668	3
KRT81	ENST00000327741	chr12	52679697	52685318	1
LAPTM4A	ENST00000175091	chr2	20232411	20251789	2
NUCKS1	ENST00000367142	chr1	2.06E+08	205719404	2
LAPTM4B	ENST00000445593	chr8	98787285	98865241	3
PERP	ENST00000421351	chr6	1.38E+08	138428648	1
KRT19	ENST00000361566	chr17	39679869	39684560	7

Supplementary Table S4: The number highly abundant 10 transcripts across experimental conditions.

	**siAKT1 (20)**	**Inhibitor VIII(22)**	**siAKT1 EGF (1550)**	**Inhibitor EGF (71)**
Protein_coding	13	8	1432	61
LincRNA	3	5	40	1
Antisense	2	3	39	4
Sense_overlapping			6	
Sense_intronic			5	1
Pseudogene	2	2	17	2
Processed_transcript		3	9	1
misc_RNA			1	
Polymorphic_pseudogene			1	
snoRNA		1	1	
TR_V_gene				1

Supplementary Table S5: The number of potential differentially expressed genes according to their coding potential.

Transcripts	siAKT1	Inhibitor VIII	Gene Symbol	Functional Involvement	Ref.
ENSG00000004059+ ENSG00000106328:E002	-19.12	0.02	ARF5, FSCN3	FSCN3 involved in cancer metastasis	[68]
ENSG00000244274+ ENSG00000204070+ ENSG00000254806:E034	-18.79	-0.16	DBNDD2, SYS1, SYS1-DBNDD2	DBNDD2 upregulated in BRCA1 mutated Breast cancer, SYS1 mutation found in cancer	[63,86]
ENSG00000244274+ ENSG00000204070+ ENSG00000254806:E033	-18.72	-0.13			
ENSG00000067836:E047	-17.94	-0.33	ROGDI	Found as target in Cervical cancer	[70]
ENSG00000067836:E043	-17.88	0.18			
ENSG00000067836:E046	-17.73	-0.07			
ENSG00000067836:E045	-17.73	-0.03			
ENSG00000067836:E044	-17.73	0.04			
ENSG00000112977:E012	-17.93	0.51	DAP	Death associated protein inactivation reported in many Breast cancers	[87]
ENSG00000100605:E027	-17.92	0.41	ITPK1	Over expressed in Breast cancer	[69]

Supplementary Table S6: The 10 selected splice variation alterations by each silencing method used in the experiment compared to the control.

Transcripts	siAKT 1 + EGF	Inhibitor VIII + EGF	Gene Symbol	Functional involvement	Ref.
ENSG00000075292:E053	-28.16	1.48	ZNF638	Colorectal cancer	[88]
ENSG00000123636+ ENSG00000225369:E048	-26.96	1.92	BAZ2B , NSG00000225369 Gene	No reported cancer relationship	
ENSG00000116001+ ENSG00000115998:E061	-22.43	1.24	TIA1, C2orf42	TIA1 found decreased in human cancer	[71]
ENSG00000156011+ ENSG00000244018:E018	-19.09	-1.44	PSD3 , RPL35P6	PSD3 found downregulated in Breast cancer	[72]
ENSG00000155903:E036	-17.09	-1.15	RASA2	Mutation is high risk in Breast cancer	[89]
ENSG00000104835+ ENSG00000269190+ ENSG00000269547:E057	-16.69	-0.81	SARS2, FBXO17	FBXO17 promotes cancer in adenocarcinoma	[90]
ENSG00000145390:E030	-16.87	0.19	USP53	Decreased USP53 levels are a reliable marker of lung adenocarcinoma	[92]
ENSG00000188739+ ENSG00000054267:E064	-17.58	-0.67	RBM34, ARID4B	ARID4B is a potential preogression modifier gene	[93]
ENSG00000188739+ ENSG00000054267:E066	-17.04	-1.00			
ENSG00000120370:E004	1.28	14.94	GORAB	Reported co amplified in cancer	[77]

Supplementary Table S7: The select splice variations identified in different experimental conditions.

A.

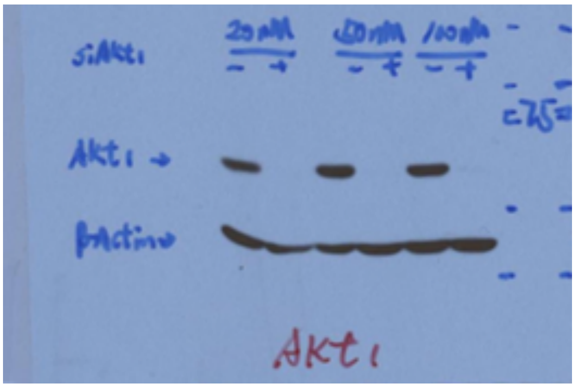

siAKT1

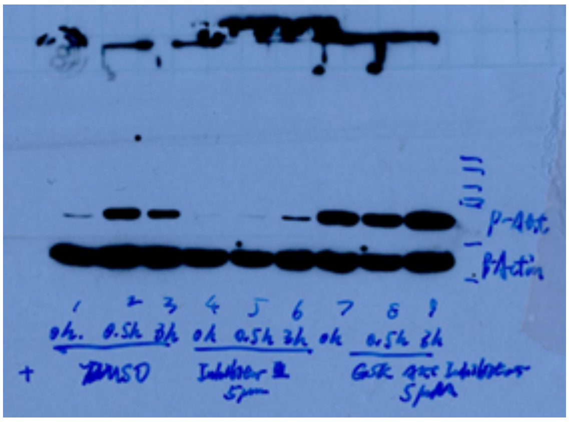

Inhibitor VIII, lanes 4-6

B.

-EGF

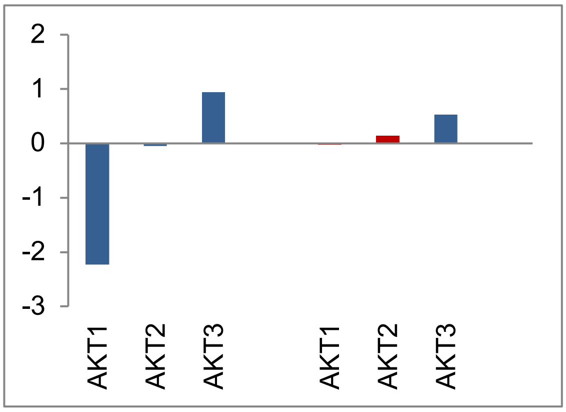

siAKT1 Inhibitor VIII

+EGF

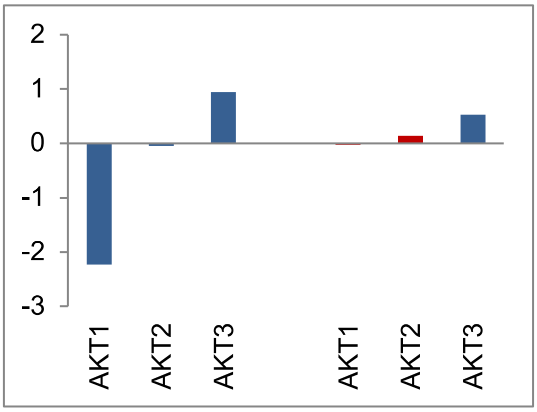

siAKT1 Inhibitor VIII

Figure S1: Expression levels of AKTs. (**A**) Levels of AKT1 and Actin in MCF-7 cells after silencing siAKT1 using for different concentrations for 36 hr (left panel) or using AKT Inhibitor VIII (right panel, lanes 4-6) or an unrelated control inhibitor (right panel, lanes 7-9). Western blotting was performed by AKT1 and Actin antibodies. (**B**) The levels of AKT1 expression after silencing is shown using the processed datasets from RNA-Sequencing experiments.

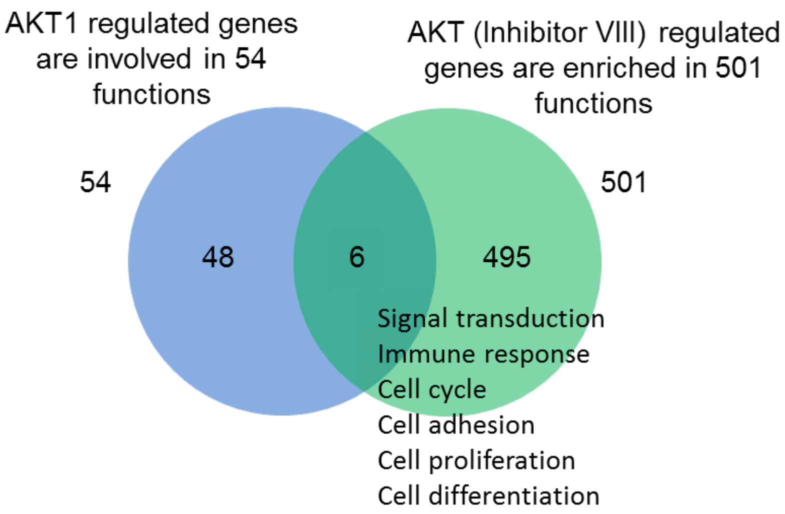

Figure S2: Comparative analysis of functions regulated by AKT1 regulated genes versus the functions inhibited by pan- AKT inhibitor VIII, showing six predicted shared functions.

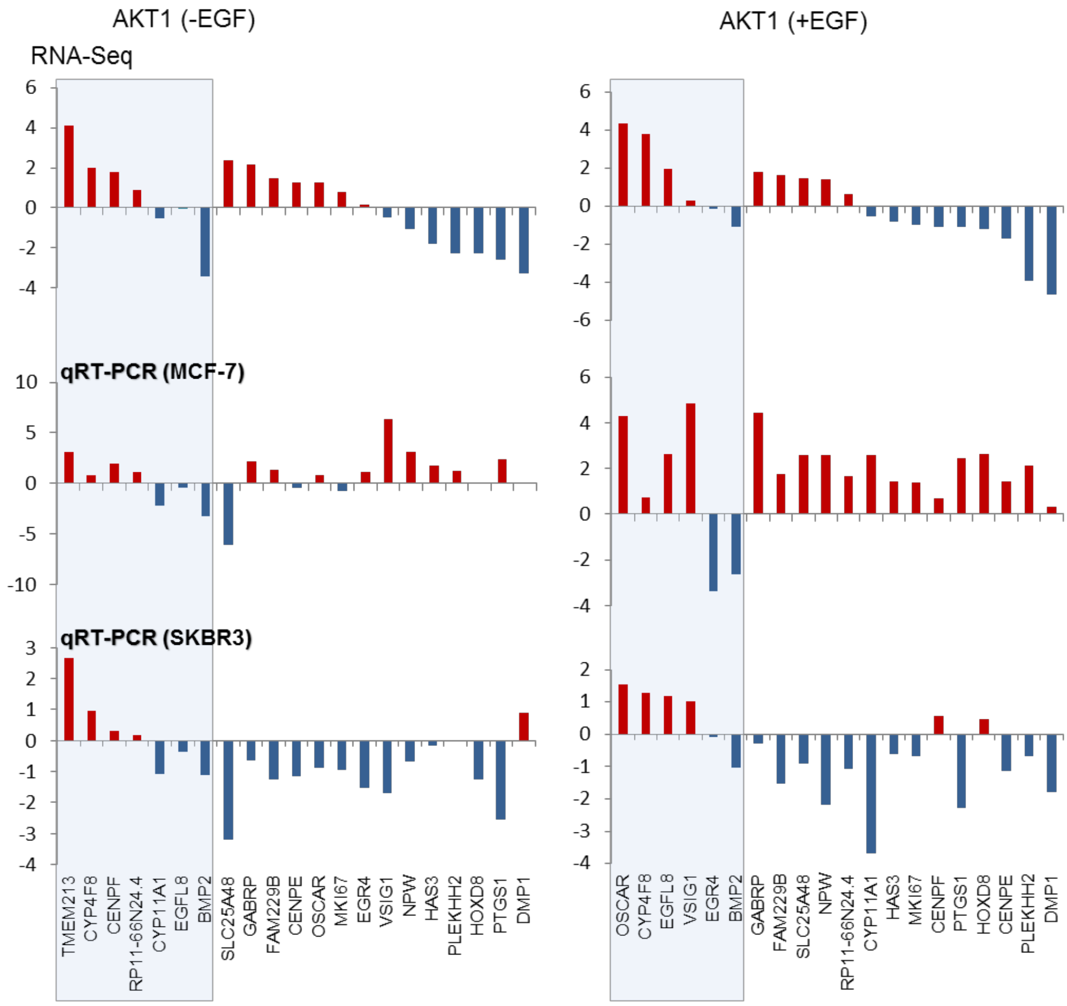

Figure S3: Status of the fold-change values from the RNA-Sequencing and RT-PCR assays for selected genes using MCF-7 and SKBR3 cell lines. Genes follow similar pattern of expression were connected with the box and the upregulated genes are indicated with red and down regulated genes are indicated with blue.

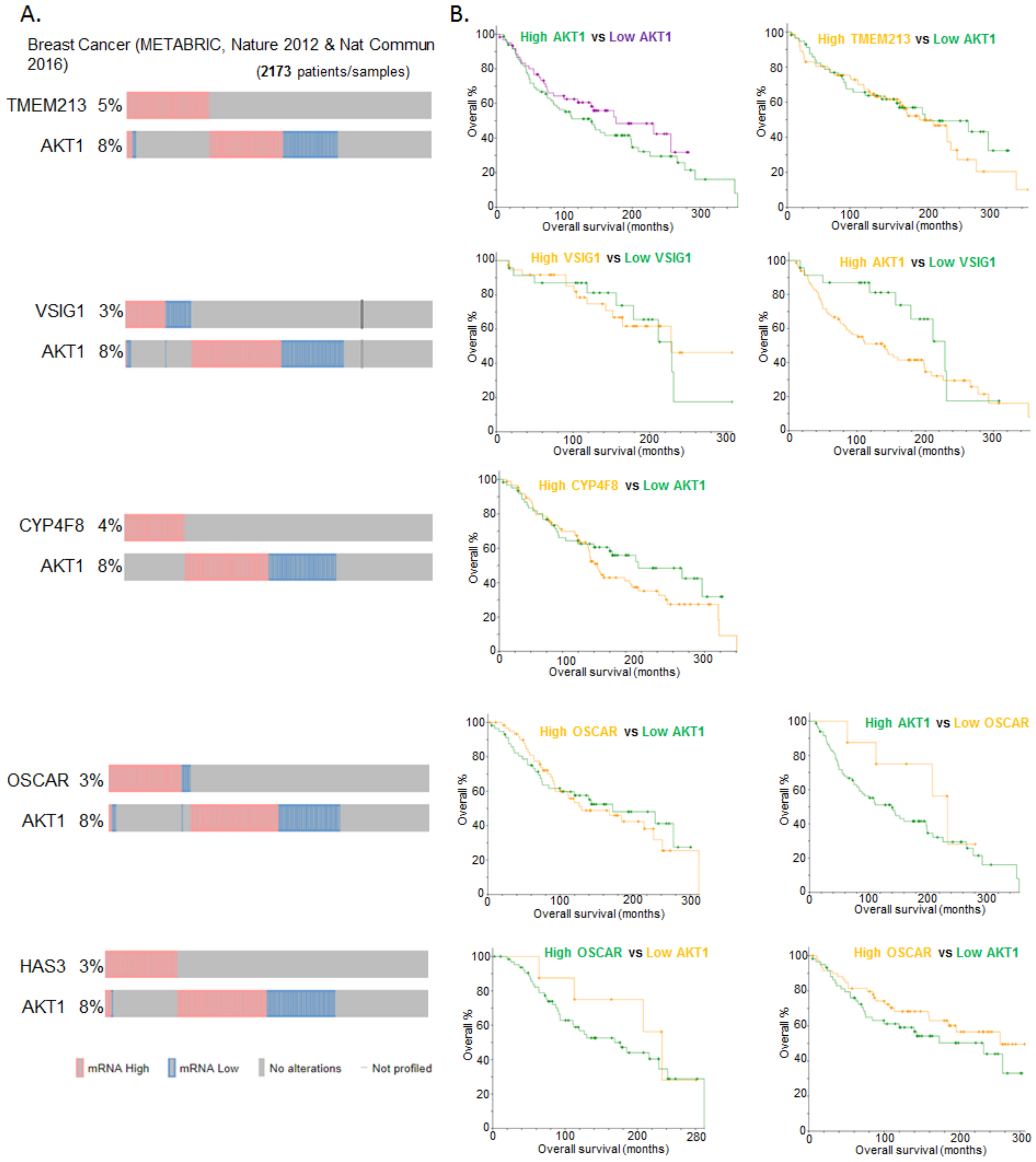

Figure S4: The expression levels (**A**) of selected genes in breast cancer dataset along with the changes in the patient survival (**B**).

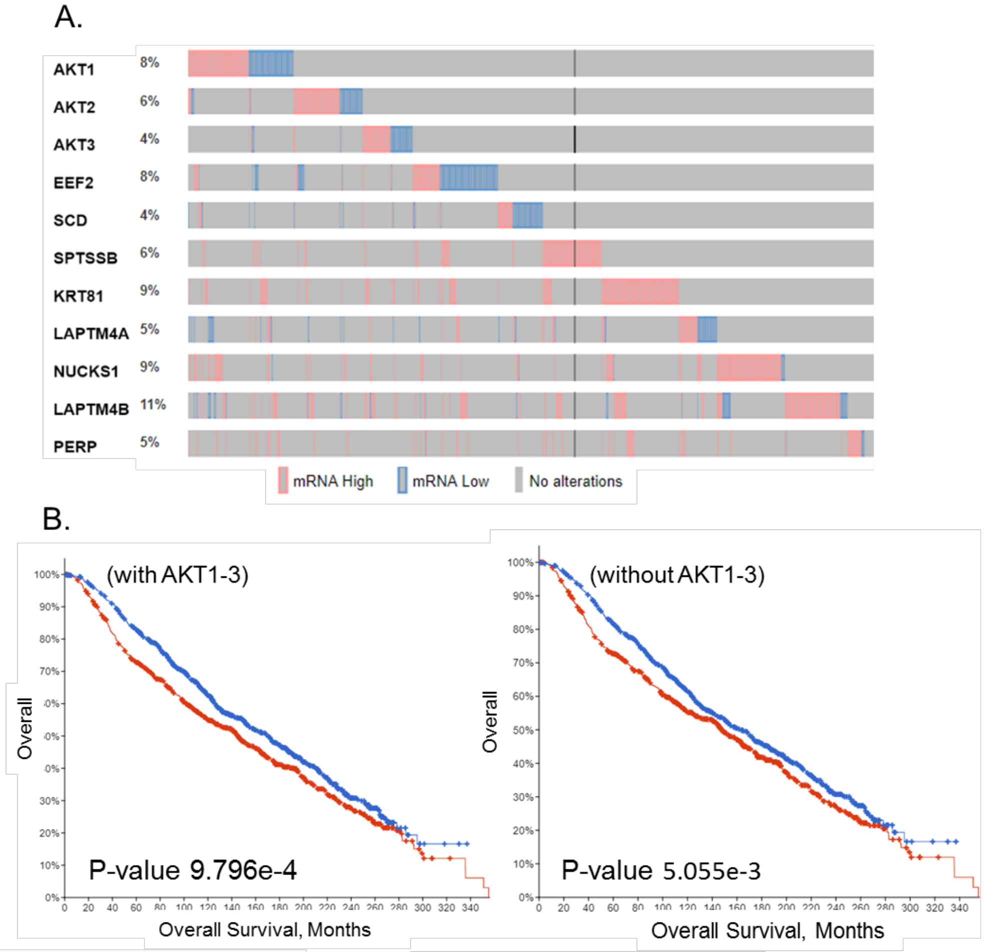

	Number of Cases	Number of Events	Median Months Overall (95% CI)	Number of Cases	Number of Events	Median Months Overall (95% CI)
Altered group	919	569	144.97 (129.23 - 159.23)	771	477	147.37 (130.87 - 163.70)
Unaltered group	985	534	170.63 (153.83 - 184.70)	1133	626	163.20 (150.10 - 174.50)

Figure S5: Gene expression and survival analysis of select highly abundant genes. (**A**) The levels of expression of AKT1-3 isoforms and highly abundant genes in Breast Cancer (METABRIC, Nature 2012 & Nat Commun 2016) data [49-51] from CBioPortal [52, 53] . (**B**) Overall patient survival status based on the alterations for highly abundant genes and also along with AKT isoforms.
